# The Superficial Temporal Artery and Zygomatico-Orbital Artery: Superficial Arterial Distribution of the Anterior Temple Area

**DOI:** 10.1155/2022/3790546

**Published:** 2022-05-26

**Authors:** Hyun Jin Park, Ji-Hyun Lee, Wonsug Jung

**Affiliations:** ^1^Division in Anatomy and Developmental Biology, Department of Oral Biology, Human Identification Research Institute, BK21 FOUR Project, Yonsei University College of Dentistry, Seoul, Republic of Korea; ^2^Department of Anatomy, Yonsei University Medical College, 50-1 Yonsei-ro, Seodaemun-gu, Seoul, Republic of Korea

## Abstract

A hollow temple may give rise to a false impression of early facial aging. This is corrected with dermal fillers that are injected into the hollow temple area to produce a smoother facial contour. However, various complications of this procedure have been reported, with the most common being the inadvertent injection of the filler material into the superficial temporal artery (STA). The aim of this study was to investigate the topographic anatomy of the STA and zygomatico-orbital artery (ZOA) to provide essential anatomical information to aid in various clinical procedures involving the temporal region. The superficial arterial distribution of the temple area was studied in 43 hemisectioned Korean cadavers. The courses of the STA and ZOA were identified and classified based on the line connecting the tragus and the superciliary arch (TR-SA line). The ZOA was present in 85.2% of cases and bifurcated from the frontal branch of the STA, after which it ran along the TR-SA line. In this study, the STA pattern was classified into a typical pattern where the ZOA coexists with the STA and a lower pattern where the ZOA was absent. The current findings suggested that the ZOA ran close to the TR-SA line. Therefore, to minimize vascular complications during invasive procedures, injection into this area should be avoided. In addition, clinicians should verify the existence of ZOA and the course of STA before performing various clinical procedures.

## 1. Introduction

The term “hollow temple” implies a decrease in the volume of soft tissue overlying the temporal region due to the loss of subcutaneous tissues or temporalis muscle atrophy. This defect is treated with temporal fossa augmentation performed using filler injections [1]. As interest in beauty increased, the number of temporal fossa augmentation also increased. Consequently, the number of reconstructive flap surgeries for supplying blood circulation to defective areas of the face or neck increases [3]. However, such procedures may be associated with various complications such as hematoma, headache, ophthalmoplegia, ptosis, embolism, and even blindness as a result of accidental injection of the filler material into the arteries of the temporal region [2]. In order to proceed with a safer and more successful filler injection or flap surgery, it is of extreme importance to have a thorough knowledge of the arterial supply of the temporal region. For this reason, various studies on the vascular anatomy of the temporal region have been conducted [3–7].

STA is the main artery that supplies blood to the temporal and parietal regions. The STA is a terminal branch of the external carotid artery, which arises from the depths and runs upwards, gradually taking a superficial course. On average about 4 cm above the zygomatic process of the temporal bone, the STA divides into the frontal and parietal branches; thus, the branches of the STA run superficially and terminate in the subcutaneous layer [8–10]. For clinical reasons, many studies have been conducted to understand the anatomical variations of STA such as the shape, branching patterns, and location of the STA. In addition, the frontal branch of STA (STA-Fbr) has been studied extensively, which is the most important blood vessel in clinical procedures such as the temporal fossa augmentation [4, 6, 11–14].

Recently, greater emphasis has been placed on the zygomatico-orbital artery (ZOA) which is another major artery in the temporal region [3]. ZOA bifurcates from the STA and may occasionally arise from the frontal, middle temporal, or parietal branches of the STA and has been reported to be present in 78% to 92% of specimens in previous studies [9]. Also, Bracco et al. reported that the ZOA may be connected to the supraorbital artery and the lacrimal artery [15]. Therefore, in-depth understanding of the anatomical considerations in relation to the ZOA is needed due to the increasing number of reconstructive and cosmetic surgeries in the temporal region. However, detailed topographic anatomical studies of ZOA are rare [3, 8, 16]. Although ZOA is closely related to STA because it arises from STA, the correlation between STA and ZOA has not yet been investigated.

The aim of this study was to investigate the topographic anatomy of the frontal branch of the STA (STA-Fbr) and the ZOA and furthermore to help clinicians perform clinical procedures with increased precision in the temporal region by proposing an easy, reliable, and predictable method of locating the ZOA using the surface anatomy.

## 2. Materials and Methods

### 2.1. Cadaveric Dissection

Fifty-four hemifaces (19 right sides and 24 left sides) from 43 embalmed Korean cadavers (30 males and 13 females; mean age, 72.83 [32-97] years) were used in this study. There was no history of trauma or surgery of the temple area. Using the hairline (HL) as a landmark, the skin and subcutaneous tissue below it were carefully dissected to expose the arteries of the temporal region. This was followed by examination of the relationship between the arteries of the temple area and the HL. The remaining area was then carefully dissected.

### 2.2. Topographic Analysis of the Course of the Arteries

**Figure 1:**
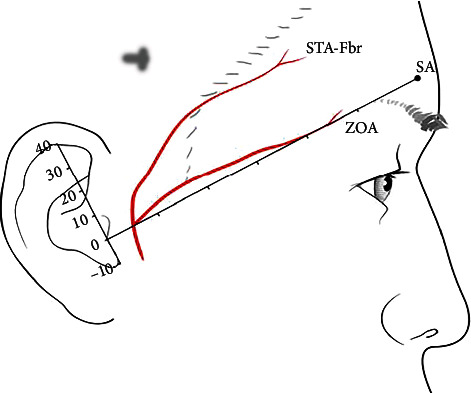
Reference lines and points for measuring the superficial temporal artery (STA-Fbr) and zygomatico-orbital artery (ZOA). TR: tragus; SA: superciliary arch; unit: mm.

After dissection, the presence of ZOA and the relationship between STA and ZOA were confirmed, along with the origin and location of both the arteries. Topographic analysis of the courses of the ZOA and STA-Fbr was performed based on the line connecting two facial landmarks, the tragus (TR) and the superciliary arch (SA). The distances to each artery were measured from the perpendicular to the TR-SA line at every 1 cm interval along this perpendicular (Figure 1). Then, in 15 specimens, the location of the origin of the ZOA, which bifurcated from the STA, was also analyzed based on the TR-SA line. All measurements were conducted using digital calipers (CD-15APX; Mitutoyo Corporation, Japan).

All authors were well informed of the WMA Declaration of Helsinki—Ethical Principles for Medical Research Involving Human Subjects—and confirmed that the present study firmly fulfilled the declaration. None of the authors have financial or private relationships with commercial, academic, or political organizations or people that could have improperly influenced this research.

## 3. Results

### 3.1. Topographic Analysis of the ZOA

**Figure 2:**
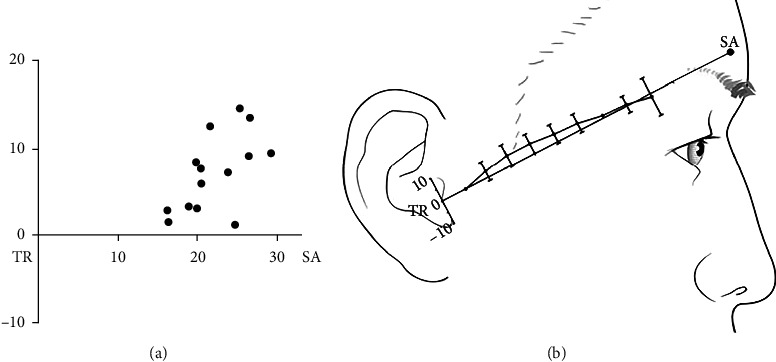
Topographic analysis of the zygomatico-orbital artery (ZOA). (a) The origin of the ZOA is generally located above the line connecting the tragus (TR) and superciliary arch (SA). (b) ZOA runs within 1 cm of the TR-SA line. Range indicates SD. TR: tragus; SA: superciliary arch; unit: mm.

The ZOA was present in 46 of 54 hemifaces (85.2%) and bifurcated from the frontal branch of the STA in all cases. On average, the origin of the ZOA along the TR-SA line was located 7.0 ± 4.8 mm above and 22.4 ± 4.2 mm ahead of the TR (Figure 2(a)). On average, ZOA ran within 5 mm along the TR-SA line, and it was located at the highest point of 4 cm (4.41 mm + 4.2 mm) and the lowest point of 9 cm (−1.24 mm + 8.04 mm) from the TR. Thus, in all cases where the ZOA was present, the ZOA ran within 1 cm of the TR-SA line (Figure 2(b)).

### 3.2. Topographic Analysis of the STA-Fbr

**Figure 3:**
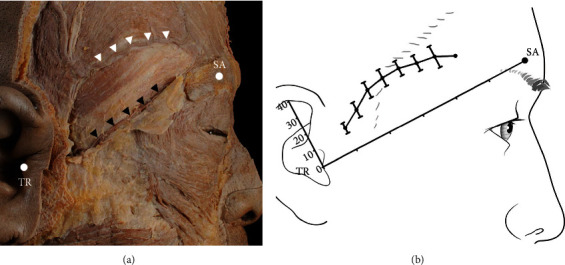
Typical pattern of the frontal branch of the superficial temporal artery (STA-Fbr). (a) Pictorial representation of the typical STA-Fbr with the ZOA. The STA-Fbr runs to the forehead above the ZOA. White arrows indicate STA-Fbr typical pattern (type I). Black arrows indicate ZOA. (b) The typical pattern of the STA-Fbr runs to the forehead in the form of an arch along the TR-SA line. The STA-Fbr is located farthest from the TR at 5 cm. TR: tragus; SA: superciliary arch.

**Figure 4:**
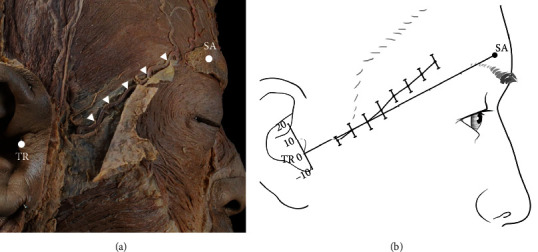
Lower pattern of the frontal branch of the superficial temporal artery (STA-Fbr). (a) Pictorial representation of the lower pattern of STA-Fbr without the ZOA. The STA-Fbr runs to the forehead. White arrows indicate the STA-Fbr lower pattern (type II). (b) The lower pattern of the STA-Fbr was located near to the TR-SA line. TR: tragus; SA: superciliary arch.

According to the presence of the ZOA, the course of the STA-Fbr differed significantly. STA-Fbr showed a typical pattern in the presence of ZOA (Figure 3(a)). However, when the ZOA was not present, the STA-Fbr ran at a lower level than the typical pattern (Figure 4(a)). Based on the level of the STA-Fbr relative to the ZOA, the STA-Fbr was classified into two types: type I (typical pattern), where the ZOA coexists with the STA-Fbr, and type II (lower pattern), where the ZOA does not coexist with the STA-Fbr. The average locations of the typical type of STA-Fbr varied depending on the measurement point, but generally it ran 2 cm above the TR-SA line. The STA-Fbr was closest to the TR-SA line at a point 1 cm from the TR (13.03 mm ± 3.09 mm) and farthest at a point 5 cm from the TR (28.49 mm ± 4.25 mm) (Figure 3(b)). The lower type of STA-Fbr was located nearer to the TR-SA line.

During the dissection, it was noted that the STA-Fbr was lower than the TR-SA line at a point 1 cm above from the TR (−2.02 mm + 2.99 mm), and then, it ran upward as it went forward, so at a point 9 cm above the TR, it was located far away from TR-SA line (12.61 mm ± 4.33 mm) (Figure 4(b)).

## 4. Discussion

The precise location of ZOA that supplies blood to the skin of the lateral canthal and suprazygomatic territory [17] is an anatomically important consideration during temporal fossa augmentation. Although many vascular complications associated with ZOA have been reported to occur during temporal fossa augmentation [18–21], detailed topographic analysis of the ZOA is rare in the current literature. In a previous study, the ZOA was found in 78% to 92% of individuals and originated from the frontal, middle, or parietal branches of the STA [9]. Additionally, the ZOA has been described as running along and parallel to the upper border of the zygomatic arch [17]. We examined and studied the ZOA with respect to its anatomical course, surface landmarks, and the relationship with other structures, including the STA-Fbr.

In this study, similar to the findings of a previous study, the ZOA existed in 85% of the evaluated cases and average bifurcation point of the ZOA was located 22.4 ± 4.2 mm above and 7.0 ± 4.8 mm ahead of the TR. In contrast, the ZOA did not run along or parallel to the zygomatic arch. The results of this study show that the ZOA ran along the TR-SA line within ±1 cm.

STA-Fbr is considered to be the most important artery for various clinical procedures in the temporal region, and many studies have been conducted, including morphological classification of STA-Fbr. In a previous study, Lei et al. [22] divided STA into high and low types and reported that they were present in 64% and 36%, respectively. Further, Shin et al. [23] reported that high, intermediate, and low types were present in 73%, 20%, and 7%, respectively. As such, previous studies classified the pattern of STA-Fbr; however, detailed measurement of the arterial path and information about the relationship of ZOA was lacking.

In the present study, we found that the pattern of STA-Fbr is different depending on the existence of ZOA. Accordingly, the representative pattern of STA-Fbr in which ZOA exists is classified into a typical type, and in the absence of ZOA, an atypical pattern was classified as low type. In addition, the position was measured according to each shape. These are corresponding to the high type and low type of the previous study, respectively [22, 23]. Furthermore, we speculated that due to an embryological compensation for the absence of the ZOA, STA-Fbr exists in a lower position.

We further examined the relationship of the ZOA with the HL as there are no reports confirming the relationship between HL and blood vessels in the temporal region. Both the typical STA-Fbr and ZOA were covered with the HL. In particular, type II STA-Fbr and the ZOA ran from the origin, straight through the sideburn, and were located lower than the level of HL.

**Figure 5:**
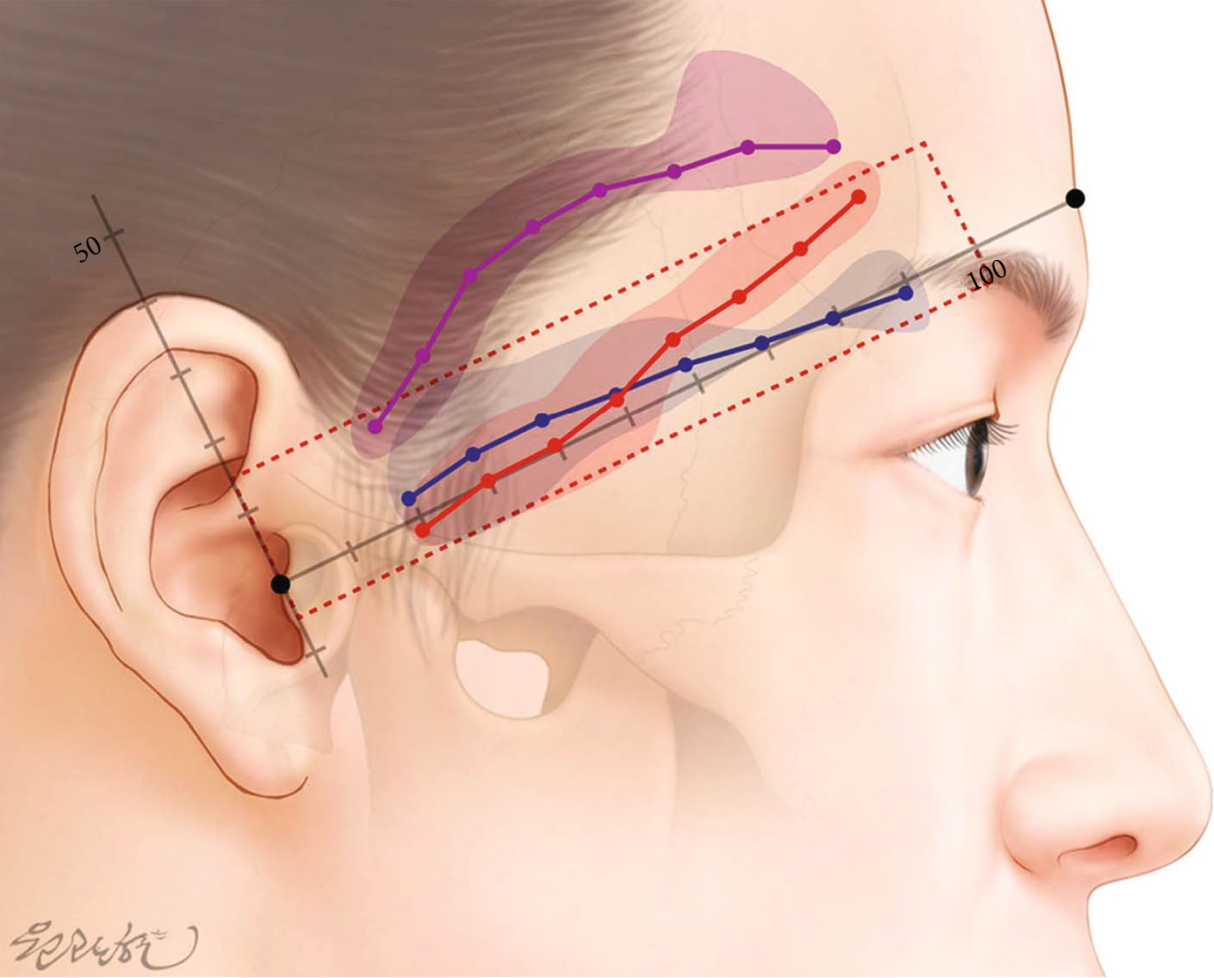
Illustration of the STA-Fbr and ZOA according to the measurement result. Purple: STA-Fbr typical pattern (type I); red: STA-Fbr lower pattern (type II); blue: ZOA.

The results of this study may provide important information to clinicians in performing a successful temple augmentation. The temporal region, which is not covered by a sideburn, is primarily involved in cosmetic procedures routinely. However, ZOA or type II STA-Fbr is frequently found within 1 cm of the danger line (TR-SA line), making this area unsuitable as an entry site for filler injection (Figure 5). Therefore, to minimize complications during injection, this area should be avoided. In cases where injection in this area is unavoidable, it should be injected gradually with a cannula and regurgitated.

In addition, the temporoparietal, cheek rotation, cervicofacial, or forehead flaps are conducted for facial reconstruction using the superficial temporal, transverse facial, zygomatico-orbital, and zygomatico-facial arteries [14, 24]. During reconstructive flap surgeries with the ZOA, sacrifice of the superficial temporal artery can be avoided. Consequently, it can preserve the microcirculation of the temporal region. If necessary, the preserved STA can be used as a flap, and in case the STA is ruptured, it can be replaced with ZOA. Therefore, the results of this study suggested that when conducting reconstructive flap surgeries, the ZOA flap can be used more easily while avoiding damage to the STA.

This study had certain limitations; for example, the study was conducted on elderly cadavers that belonged to a single race. To overcome these limitations, we believe that additional studies on living specimens of various races and age groups are needed. In addition, the relationship with more diverse blood vessels in the temporal region, such as the middle temporal vein or deep temporal artery, also needs to be studied in depth.

Moreover, although our study confirmed the relationship between HL and blood vessels for the first time, we could not proceed with an in-depth study on the difference in the shape of HL and its relationship with blood vessels. The HL differs according to individual, gender, and races. In particular, because the shape of HL varies greatly depending on the presence or absence of hair loss and shape of sideburns [25, 26], more detailed research is needed.

The finding of the current study showed that about 85% of ZOA has been identified, and it can be easily identified using an ultrasound device at the TR-SA line region, which can help prevent complications during other nonsurgical and surgical procedures as well, such as botulinum toxin injection, surgery, and flap.

## Data Availability

The measurement of the ZOA data used to support the findings of this study is available from the corresponding author upon request.
